# Leisure-Time Physical Activity, but not Commuting Physical Activity, is Associated with Cardiovascular Risk among ELSA-Brasil Participants

**DOI:** 10.5935/abc.20170178

**Published:** 2018-01

**Authors:** Francisco José Gondim Pitanga, Sheila M.A. Matos, Maria da Conceição Almeida, Sandhi Maria Barreto, Estela M. L. Aquino

**Affiliations:** 1 Universidade Federal da Bahia (UFBA), Salvador, BA, Brazil; 2 Centro de Pesquisas Gonçalo Moniz da Fundação Oswaldo Cruz (FIOCRUZ), Salvador, BA, Brazil; 3 Universidade Federal de Minas Gerais (UFMG), Belo Horizonte, MG, Brazil

**Keywords:** Exercise, Exercise Movement Techniques, Risk Factors, Cardiovascular Diseases / prevention & control, Epidemiology

## Abstract

**Background:**

Despite reports in the literature that both leisure-time physical activity (LTPA) and commuting physical activity (CPA) can promote health benefits, the literature lacks studies comparing the associations of these domains of physical activity with cardiovascular risk scores.

**Objective:**

To investigate the association between LTPA and CPA with different cardiovascular risk scores in the cohort of the Longitudinal Study of Adult Health ELSA-Brasil.

**Methods:**

Cross-sectional study with data from 13,721 participants of both genders, aged 35-74 years, free of cardiovascular disease, from ELSA Brazil. Physical activity was measured using the International Physical Activity Questionnaire (IPAQ). Five cardiovascular risk scores were used: Framingham score - coronary heart disease (cholesterol); Framingham score - coronary heart disease (LDL-C); Framingham score - cardiovascular disease (cholesterol); Framingham score - cardiovascular disease (body mass index, BMI); and pooled cohort equations for atherosclerotic cardiovascular disease (ASCVD). Associations adjusted for confounding variables between physical activity and different cardiovascular risk scores were analyzed by logistic regression. Confidence interval of 95% (95%CI) was considered.

**Results:**

LTPA is inversely associated with almost all cardiovascular risk scores analyzed, while CPA shows no statistically significant association with any of them. Dose-response effect in association between LTPA and cardiovascular risk scores was also found, especially in men.

**Conclusions:**

LTPA was shown to be associated with the cardiovascular risk scores analyzed, but CPA not. The amount of physical activity (duration and intensity) was more significantly associated, especially in men, with cardiovascular risk scores in ELSA-Brasil.

## Introduction

Physical activity (PA) is inversely associated with all-cause mortality, especially with cardiovascular mortality.^1,2^ Several studies have shown that PA, especially when considered in leisure-time domain, has a protective effect against chronic diseases and cardiovascular risk factors, including diabetes, dyslipidemia, hypertension and inflammatory markers.^3-7^

Cardiovascular risk scores are algorithms that have been proposed to stratify coronary and/or cardiovascular risks in order to estimate the probability of developing such diseases in ten years from the calculation in a given population. The first to be developed was presented by Wilson et al.^8^, focusing on coronary artery disease risk and based on the Framingham score. Afterwards, D'Agostino et al.^9^ developed an assessment tool that would allow the identification of patients at high risk for all and any initial atherosclerotic event in ten years from the test application (coronary artery disease, cerebrovascular diseases, peripheral vascular disease, and heart failure) by means of measures readily available in clinical practice. More recently, the American College of Cardiology (ACC) and the American Heart Association (AHA)^10^ suggested new pooled equations to assess the risk of atherosclerotic cardiovascular diseases (within ten years), defined as the first occurrence of nonfatal myocardial infarction, death from coronary artery disease, and fatal/nonfatal stroke.

Despite reports in the literature that both leisure-time physical activity (LTPA)^11,12^ and commuting physical activity (CPA)^13^ can benefit health, there is a lack of studies analyzing and comparing the association of both PA domains with cardiovascular risk scores.^14^ The main explanations for associations found between PA and cardiovascular risk scores are related to the favorable changes caused that PA causes in blood pressure, lipid profile, and glycemic levels.^15-17^

Establishing a quantitative relation between LTPA and/or CPA with cardiovascular risk scores can help public health authorities in best spreading messages that encourage the society to practice physical activities.

The purpose of this paper was to verify the association between LTPA and/or CPA with different cardiovascular risk scores in the cohort from Longitudinal Study of Adult Health (ELSA-Brasil).

## Methods

### Population and sample

ELSA-Brasil is a cohort study of 15,105 economically active or retired people of both genders, aged 35-74, from six teaching and research institutions in the cities of Salvador, Vitória, Belo Horizonte, Rio de Janeiro, São Paulo, and Porto Alegre, whose methodological details have been previously described.^18,19^ For the present study, all baseline participants (2008-2010) who answered the questionnaires about PA were selected, as long as they had the information required to calculate cardiovascular risk scores. After excluding participants who reported previous myocardial infarction, stroke, peripheral vascular disease, and heart failure, the sample was formed with 13,721 participants (45.3% males, 54.7% females).

ELSA-Brasil was approved by the National Commission for Research Ethics (CONEP) and by all Ethics Committees of the research centers involved. All participants signed the informed consent form, assuring secrecy and confidentiality to data.

### Data production

Data were collected by a team of interviewers and trained evaluators, all of them certified by a quality control committee^19^ and able to carry out the study protocol at the ELSA-Brasil Research Center. Face-to-face interviews were conducted with standardized and previously validated questionnaires.

Evaluation of physical activity

The International Physical Activity Questionnaire (IPAQ) was applied to identify and quantify PA, consisting of questions about the frequency and duration of physical activities at work (moderate and vigorous walking), while commuting, in domestic activities, and in leisure time.^20^ ELSA-Brasil only addressed leisure time and commuting activities. PA was measured in minutes per week by multiplying weekly frequency by each event’s duration of each.

For the purpose of this study, participants were classified as to leisure-time activities as follows:

- sedentary (< 10 min/week, any PA);
- (≥ 10 min to < 150 min/week of walking, moderate PA and/or 10 min to < 60 min/week of vigorous PA and/or 10 min to < 150 min/week of any combination of walking, moderate and vigorous PA);
- physically active (≥ 150 min/week of walking, moderate PA and/or ≥ 60 min/week of vigorous PA and/or ≥ 150 min/week of any combination of walking, moderate and vigorous PA);
- very active (≥ 150 min/week of vigorous PA, or ≥ 60 min/week of vigorous PA plus 150 min/week of any combination of walking and moderate PA).

For dichotomized analyzes, participants sorted as sedentary and not very active were considered insufficiently active, and active participants were those sorted as physically active and very active.

Commuting PA was categorized as insufficiently active (< 150 min/week of walking and/or cycling) and physically active (150 min/week of walking and/or cycling).

Evaluation of cardiovascular risk

Five cardiovascular risk scores were used. Two of them were proposed by Wilson et al.^8^ and aimed to estimate the risk of coronary artery disease. Variables used were: age, systolic and diastolic blood pressure (BP), high-density lipoprotein (HDL-C), diabetes, smoking, and total cholesterol in the first; and age, systolic and diastolic BP, HDL-C, diabetes, smoking and low-density lipoprotein (LDL-C) in the second. The third and fourth scores, proposed by D'Agostino et al.,^9^ aimed to identify patients at high risk for any initial atherosclerotic event (coronary heart disease, cerebrovascular diseases, peripheral vascular disease, and heart failure), using following variables: age, treated and untreated systolic and diastolic BP, HDL-C, body mass index (BMI), diabetes, smoking, and total cholesterol in the third; and age, treated and untreated systolic and diastolic BP, HDL-C, diabetes, smoking, and BMI in the fourth. The fifth score, indicated by ACC and AHA,^10^ aimed at estimating the risk for atherosclerotic diseases. The variables used were: age, treated and non-treated systolic BP, total cholesterol, HDL-C, smoking, and diabetes. All cardiovascular risk scores were calculated for ELSA-Brasil participants, with detailed scoring scheme previously reported.^8-10^ Participants with scores ≥ 20% were considered at high risk for future cardiovascular events.^21^

### Evaluation of covariables

BP was obtained with a validated oscillometric device (Omron HEM-705CPINT) after a five-minute rest, with the subject sitting in a quiet and temperature-controlled room (20-24°C). Three measurements were taken at 1-min intervals each. The mean of the last two BP measurements was calculated and used in our analysis.

Definition of diabetes was based on self-reported information and laboratory exams. Patients were considered to have been diagnosed if they had been previously informed by a physician that they had diabetes or if they had used medication for diabetes in the last two weeks. Patients not previously diagnosed with diabetes were classified as having diabetes when fasting plasma glucose level was ≥ 7.0 mmol/L, two-hour post-load glucose was ≥ 11.1 mmol/L, or glycated hemoglobin (HbA1c) was ≥ 6.5%.^22,23^ Participants were sorted as hypertensive if systolic blood pressure (SBP) was ≥ 140 mmHg, diastolic blood pressure (DBP) was ≥ 90 mmHg or if they had taken any medication to treat hypertension in the last two weeks.

Total cholesterol and HDL-C were determined by the enzymatic colorimetric method. LDL-C was calculated by the Friedewald equation.

Obesity was identified by BMI, being applied the equation BMI = weight(kg)/height(m)^2^ and adopted the following cutoff point: obesity = 0 if BMI <30.0 and obesity = 1 if BMI ≥ 30.0.

### Data analysis procedures

Descriptive measures (proportions) were calculated for all categorized variables. Analyzes were stratified by gender at first. The differences between men and women as to variables were identified by the chi-square test. Associations between dependent (different cardiovascular risk scores) and independent variables (LTPA and CPA) were analyzed by logistic regression. The following were considered as potential confounding variables: age, obesity, family income, educational level, and functional status. Variables presenting simultaneous evaluation (tetrameric matrix) of correlation coefficient rho < 0.60 and p ≤ 0.05 upon bivariate analysis were selected as model.

Analysis of confounding variables was made by comparing Odds Ratio (OR) of the crude association and adjusted association for possible confounders. The parameter used to identify the difference between associations was 10%. Then logistic regression analysis was performed, starting with the complete model and then removing each of the possible confounding variables that resulted in alteration equal to or greater than 10% in the association between LTPA/CPA and cardiovascular risk scores.^24^ The modeling process did not identify effect-modifying variables, and variables age, obesity and educational level were considered confounders for men, while only age and education were identified as confounders for women. Therefore, the best model to analyze the association between LTPA/CPA with cardiovascular risk scores was adjusted for age, obesity, and educational level for males and for age and educational level for females.

Dose-response effect was also assessed for the association between LTPA and cardiovascular risk scores. Dummy variables were created for comparison between the reference group (sedentary) and each strata of the PA variable (not very active, active, very active). The Mantel Haenszel test was used to evaluate homogeneity of OR values between variables’ strata, with a significance level set at 0.05. The confidence interval was set at 95% (95%CI), and the statistical software Stata version 12.0 was used.

## Results

A total of 6,222 men (45.3%) and 7,499 women (54.7%) were included in the study. Sample characteristics are shown in Table 1. The former were reported as higher family income, more active in free time and while commuting, with higher values for cardiovascular risk scores analyzed, while the latter were found to be more educated and more frequently obese. There was a higher percentage of retired women and no statistically significant differences between men and women as to age.

**Table 1:** Baseline sample characteristics: Longitudinal Study of Adult Health (ELSA-Brasil), 2008-2010

	Males (n = 6,222)	Females (n = 7,499)	p
**Age (years)**			
34-50	3,112 (49.3%)	3,675 (48.3%)	
51-60	1,941 (30.7%)	2,437 (32.0%)	
> 60	1,261 (20.0%)	1,500 (19.7%)	0.27
**Family income (Minimum wages)**			
Up to 2	72 (1.1%)	101 (1.3%)	
2 to 8	2,496 (39.7%)	2,968 (39.2%)	
8 to 18	2,100 (33.4%)	2,927 (38.6%)	
Above 18	1,619 (25.8%)	1,582 (20.9%)	0.00
**Education**			
Incomplete elementary school	489 (7.7%)	265 (3.5%)	
Complete elementary school	515 (8.2%)	388 (5.1%)	
Complete high school	2,116 (33.5%)	2,723 (35.7%)	
Complete higher education/post-graduation	3,194 (50.6%)	4,236 (55.7%)	0.00
**Work situation**			
Retired	879 (13.9%)	1,615 (21.2%)	
Active	5,431 (86.1%)	5,991 (78.8%)	0.00
**Obesity**			
BMI < 30 kg/m^2^	4,985 (78.9%)	5,755 (75.6%)	
BMI ≥ 30 kg/m^2^	1,329 (21.1%)	1,857 (24.4%)	0.00
**Commuting physical activity**			
Insufficiently active	3,955 (63.6%)	5,081 (67.7%)	
Active	2,267 (36.4%)	2,418 (32.3%)	0.00
**Leisure-time physical activity**			
Sedentary	2,308 (37.1%)	3,572 (47.6%)	
Insufficiently active	1,166 (18.7%)	1,366 (18.2%)	
Active	1,562 (25.1%)	1,690 (22.6%)	
Very active	1,186 (19.1%)	871 (11.6%)	0.00
**Cardiovascular risk scores**			
**Framingham score - coronary heart disease (Cholesterol)**			
Score < 20%	5,481 (86.8%)	7,484 (98.3%)	
Score ≥ 20%	833 (13.2%)	128 (1.7%)	0.00
**Framingham score - coronary heart disease (LDL-C)**			
Score < 20%	5,792 (91.7%)	7,435 (97.7%)	
Score ≥ 20%	522 (8.3%)	177 (2.3%)	0.00
**Framingham score - cardiovascular disease (cholesterol)**			
Score < 20%	4,742 (75.3%)	7,194 (94.6%)	
Score ≥ 20%	1,554 (24.7%)	408 (5.4%)	0.00
**Framingham score - cardiovascular disease (BMI)**			
Score < 20%	4,355 (69.2%)	6,997 (92.1%)	
Score ≥ 20%	1,938 (30.8%)	603 (7.9%)	0.00
**Pooled cohort equations for atherosclerotic cardiovascular disease risk**			
Score < 20%	5,480 (88.3%)	7,304 (97.1%)	
Score ≥ 20%	728 (11.7%)	219 (2.9%)	0.00

BMI: body mass index; LDL-C: low-density lipoprotein. Values for both males and females were compared with chi-square test.

The association between LTPA/CPA and cardiovascular risk scores in males and females are presented in Tables 2 and 3. LTPA is inversely associated with almost all cardiovascular risk scores analyzed, while CPA is not significantly associated with none of them. Tables 4 and 5 show us the existence of a dose-response effect in association between LTPA and cardiovascular risk scores, especially among men.

**Table 2:** Association between leisure-time or commuting physical activity and cardiovascular risk scores for males: Longitudinal Study of Adult Health (ELSA-Brasil), 2008-2010

Cardiovascular risk score	Leisure-time physical activity (n = 6,222)	Commuting physical activity (n = 6,222)
Framingham score - coronary heart disease (cholesterol)*	0.72 (0.60-0.85)	0.99 (0.84-1.19)
Framingham score - coronary heart disease (LDL-C) *	0.72 (0.58-0.88)	1.04 (0.84-1.28)
Framingham score - cardiovascular disease (cholesterol)*	0.76 (0.65-0.88)	0.97 (0.83-1.17)
Framingham score - cardiovascular disease (BMI)#	0.68 (0.59-0.79)	0.96 (0.83-1.11)
Pooled cohort equations for atherosclerotic cardiovascular disease risk*	0.78 (0.65-0.95)	0.95 (0.79-1.15)

BMI: body mass index; LDL-C: low-density lipoprotein.

* Adjusted for age, obesity, and educational level;

# Adjusted for age and educational level.

**Table 3:** Association between leisure-time or commuting physical activity and cardiovascular risk scores for females: Longitudinal Study of Adult Health (ELSA-Brasil), 2008-2010

Cardiovascular risk score	Leisure-time physical activity (n = 7,499)	Commuting physical activity (n = 7,499)
Framingham score - coronary heart disease (cholesterol)*	0.64 (0.42-0.97)	1.26 (0.87-1.82)
Framingham score - coronary heart disease (LDL-C)*	0.60 (0.42-0.86)	1.14 (0.83-1.58)
Framingham score - cardiovascular disease (cholesterol)*	0.63 (0.50-0.81)	1.13 (0.90-1.41)
Framingham score - cardiovascular disease (BMI)*	0.78 (0.64-0.96)	1.02 (0.84-1.24)
Pooled cohort equations for atherosclerotic cardiovascular disease risk*	0.85 (0.63-1.16)	0.98 (0.73-1.32)

BMI: body mass index; LDL-C: low-density lipoprotein.

* Adjusted for age and educational level.

**Table 4:** Dose-response effect in association between leisure-time physical activity and cardiovascular risk scores for females: Longitudinal Study of Adult Health (ELSA-Brasil), 2008-2010

Leisure-time physical activity*	Framingham score - coronary heart disease (cholesterol)*	Framingham score - coronary heart disease (LDL-C)*	Framingham score - cardiovascular disease (cholesterol)*	Framingham score - cardiovascular disease (BMI)#	Pooled cohort equations for atherosclerotic cardiovascular disease risk*
Sedentary	1.00	1.00	1.00	1.00	1.00
Not very active	0.86 (0.68-1.08)	1.02 (0.78-1.34)	1.08 (0.87-1.33)	0.99 (0.81-1.20)	1.03 (0.80-1.32)
Active	0.81 (0.65-0.99)	0.79 (0.61-1.03)	0.91 (0.75-1.10)	0.85 (0.71-1.02)	0.87 (0.68-1.10)
Very active	0.43 (0.32-0.58)	0.52 (0.37-0.74)	0.55 (0.43-0.69)	0.47 (0.38-0.59)	0.62 (0.46-0.84)

BMI: body mass index; LDL-C: low-density lipoprotein.

* Adjusted for age, obesity, and educational level;

# Adjusted for age and educational level.

**Table 5:** Dose-response effect in association between leisure-time physical activity and cardiovascular risk scores for females: Longitudinal Study of Adult Health (ELSA-Brasil), 2008-2010

Leisure-time physical activity*	Framingham score - coronary heart disease (cholesterol)	Framingham score - coronary heart disease (LDL-C)	Framingham score - cardiovascular disease (cholesterol)	Framingham score - cardiovascular disease (BMI)	Pooled cohort equations for atherosclerotic cardiovascular disease risk
Sedentary	1.00	1.00	1.00	1.00	1.00
Not very active	1.20 (0.76-1.91)	1.04 (0.69-1.56)	1.07 (0.80-1.43)	0.92 (0.71-1.19)	1.17 (0.80-1.72)
Active	0.71 (0.43-1.16)	0.61 (0.40-0.94)	0.70 (0.52-0.93)	0.88 (0.64-1.03)	1.01 (0.72-1.44)
Very active	0.63 (0.29-1.37)	0.64 (0.34-1.20)	0.52 (0.32-0.83)	0.67 (0.46-0.95)	0.66 (0.37-1.23)

BMI: body mass index; LDL-C: low-density lipoprotein.

* Adjusted for age and educational level.

## Discussion

This study analyzed the association between LTPA/CPA with different cardiovascular risk scores. LTPA was shown to be inversely associate with risk scores analyzed, while CPA was not. These results, especially regarding LTPA, were similar to those found among 41,053 male and female Finns when moderate or high LTPA levels among both men and women, and daily walking or cycling for work only among women were found to be associated with reduced risk for coronary events.^14^

Another study which analyzed healthy behaviors, including PA measured by accelerometry, and showed an inverse dose-response association between healthy positive behaviors and risk for atherosclerotic diseases.^25^ In our study, we also found a dose-response effect in the association between LTPA and cardiovascular risk scores, mainly for males. That is, the higher the level of PA, the lower the risk of cardiovascular events.

The dose-response effect we found in this study has been reported for a long time. Kohl,^5^ has shown, in a vast literature review, the inverse dose-response association between PA and cardiovascular events, especially coronary heart disease, in different longitudinal studies. Important to note that the classification adopted in this study had the amount of LTPA calculated based on both its duration and intensity. In other studies conducted by our research group,^11,12^ in which PA was classified by intensity alone, only moderate PA was shown to hold relation with absence of hypertension and diabetes. Thus, one can assume that increasing physical activity levels to achieve greater health benefits should be suggested, bearing in mind both their intensity and duration.

Results found in the association between LTPA and cardiovascular risk scores are expected, considering that the main variables composing scores are separately associated with LTPA. Studies have pointed out that LTPA is inversely associated with high BP levels,^7,12^ diabetes,^11,26^ lipid changes,^27^ and risk of coronary heart disease.^28^ According to our results, the associations reported in previous studies are more consistent among men than among women.^28,29^

Regarding CPA, we could not demonstrate associations with cardiovascular risk scores, although previous studies have found a relationship between this type of activity and diabetes and cardiovascular mortality in individuals with type 2 diabetes. Important to note that these associations, when it comes to mortality by cardiovascular disease, have lost significance after additional adjustments for LTPA and CPA.^30,31^ These findings most probably show that the instrument used in our study to assess PA (IPAQ) does not distinguish CPA intensity-walking or cycling, for example. Thus, if subjects’ displacement is done slowly, health benefits may not be significant.

In this sense, a recent publication with data from ELSA-Brasil reported that the association between CPA and arterial hypertension was positive in women, but not statistically significant in men, while the association between LTPA and arterial hypertension was inverse for both gender.^32^ Data from ELSA-Brasil which are unpublished yet suggest that active commuting, more common in less privileged social strata, is more likely to reflect inequalities in urban mobility across Brazilian cities than a healthy habit.

The mechanisms by which PA reduces BP, blood glucose, and lipid profile remain speculative. Recent studies have emphasized the need for further research to better understand the cellular and molecular aspects involved in the main health benefits induced by PA.^33^ Relevant evidence for PA, according to the American College of Sports Medicine (ACSM),^15^ is: a) decrease in insulin levels with consequent reduction of renal sodium retention and basal sympathetic tone; b) reduction of catecholamine release levels; c) release of vasodilator substances in circulation by skeletal muscles.

As to lipid profile, there is little information about the mechanisms responsible for the reduction of LDL-C levels and very low-density lipoprotein (VLDL-C) dosage. However, the main reason for evaluating HDL-C is the greater action of lipoprotein lipase (LPL) in response to exercise: LPL accelerates VLDL-C decomposition, thus moving triglycerides from bloodstream to muscles; This causes cholesterol and other substances to be transferred to HDL-C, thereby increasing its concentration.^16^ PA also seems to play an important role in reducing blood glucose levels because it promotes proliferation of capillaries, increasing LPL activity in the muscles - which in turn increases insulin sensitivity. In addition, higher levels of PA may increase oxidative muscle fibers, which are more sensitive to insulin and can reduce glycemia.^17^

A highlight of the study is that the sample was a cohort of volunteers consisting of public servants; although they do not represent the general population, there was a significant number of participants from six Brazilian capitals. Calculation of different cardiovascular risk scores is another strong point, for it allowed us to analyze the association between them and both LTPA and CPA.

A possible limitation of the study (memory bias) can be attributed to information about PA obtained through questionnaires, even though this is an instrument widely used in national and international studies. It is important to mention that ELSA-Brasil was a longitudinal study, therefore it is expected to incorporate a more objective measure - the accelerometry - which may increase the validity of information about PA.

## Conclusions

LTPA was shown to be associated with the cardiovascular risk scores analyzed, but CPA was not. The amount of physical activity (duration and intensity) was more significantly associated with cardiovascular risk scores in ELSA Brazil.

Our results can bring important contributions to public health, since the management of public policies that promote PA can be influenced by the knowledge about type of PA that bring more health benefits. Knowing that LTPA is associated with cardiovascular risk decrease while CPA is not should be taken to public health authorities so that actions encouraging exercises in leisure and free time can be implemented.

Important to note that, although association between CPA and cardiovascular risk was not established, active commuting - such as walking and cycling - should be encouraged in certain population groups, especially when displacement to work is made at moderate intensities. Furthermore, considering dose-response effects found, especially in men, the population should be encouraged to practice more PA to maximize health benefits.
